# Delivering a Postpartum Weight Loss Intervention via Facebook or In-Person Groups: Results From a Randomized Pilot Feasibility Trial

**DOI:** 10.2196/41545

**Published:** 2023-04-27

**Authors:** Molly E Waring, Sherry L Pagoto, Tiffany A Moore Simas, Loneke T Blackman Carr, Madison L Eamiello, Brooke A Libby, Lauren R Rudin, Grace E Heersping

**Affiliations:** 1 Department of Allied Health Sciences University of Connecticut Storrs, CT United States; 2 UConn Center for mHealth & Social Media University of Connecticut Storrs, CT United States; 3 Department of Obstetrics & Gynecology University of Massachusetts Chan Medical School/UMass Memorial Health Worcester, MA United States; 4 Department of Pediatrics University of Massachusetts Chan Medical School/UMass Memorial Health Worcester, MA United States; 5 Department of Psychiatry University of Massachusetts Chan Medical School/UMass Memorial Health Worcester, MA United States; 6 Department of Population & Quantitative Health Sciences University of Massachusetts Chan Medical School/UMass Memorial Health Worcester, MA United States; 7 Department of Nutritional Sciences University of Connecticut Storrs, CT United States

**Keywords:** postpartum weight loss, Facebook, social media, pilot study, feasibility, mobile phone

## Abstract

**Background:**

Postpartum weight retention contributes to weight gain and obesity. Remotely delivered lifestyle interventions may be able to overcome barriers to attending in-person programs during this life phase.

**Objective:**

This study aimed to conduct a randomized feasibility pilot trial of a 6-month postpartum weight loss intervention delivered via Facebook or in-person groups. Feasibility outcomes were recruitment, sustained participation, contamination, retention, and feasibility of study procedures. Percent weight loss at 6 and 12 months were exploratory outcomes.

**Methods:**

Women with overweight or obesity who were 8 weeks to 12 months post partum were randomized to receive a 6-month behavioral weight loss intervention based on the Diabetes Prevention Program lifestyle intervention via Facebook or in-person groups. Participants completed assessments at baseline, 6 months, and 12 months. Sustained participation was defined by intervention meeting attendance or visible engagement in the Facebook group. We calculated percent weight change for participants who provided weight at each follow-up.

**Results:**

Among individuals not interested in the study, 68.6% (72/105) were not interested in or could not attend in-person meetings and 2.9% (3/105) were not interested in the Facebook condition. Among individuals excluded at screening, 18.5% (36/195) were ineligible owing to reasons related to the in-person condition, 12.3% (24/195) related to the Facebook condition, and 2.6% (5/195) were unwilling to be randomized. Randomized participants (n=62) were a median of 6.1 (IQR 3.1-8.3) months post partum, with a median BMI of 31.7 (IQR 28.2-37.4) kg/m^2^. Retention was 92% (57/62) at 6 months and 94% (58/62) at 12 months. The majority (21/30, 70%) of Facebook and 31% (10/32) of in-person participants participated in the last intervention module. Half (13/26, 50%) of Facebook and 58% (15/26) of in-person participants would be likely or very likely to participate again if they had another baby, and 54% (14/26) and 70% (19/27), respectively, would be likely or very likely to recommend the program to a friend. In total, 96% (25/26) of Facebook participants reported that it was convenient or very convenient to log into the Facebook group daily compared with 7% (2/27) of in-person participants who said it was convenient or very convenient to attend group meetings each week. Average weight loss was 3.0% (SD 7.2%) in the Facebook condition and 5.4% (SD 6.8%) in the in-person condition at 6 months, and 2.8% (SD 7.4%) in the Facebook condition and 4.8% (SD 7.6%) in the in-person condition at 12 months.

**Conclusions:**

Barriers to attending in-person meetings hampered recruitment efforts and intervention participation. Although women found the Facebook group convenient and stayed engaged in the group, weight loss appeared lower. Research is needed to further develop care models for postpartum weight loss that balance accessibility with efficacy.

**Trial Registration:**

ClinicalTrials.gov, NCT03700736; https://clinicaltrials.gov/ct2/show/NCT03700736

## Introduction

### Background

Postpartum weight retention contributes to long-term weight gain and obesity among childbearing persons [1-4]. Among women in the multicenter Community Child Health Network study, a third of women with a normal weight BMI prepregnancy had overweight or obesity at 1 year post partum, and 44% of women with overweight prepregnancy transitioned to obesity by 1 year post partum [1]. Among women enrolled in the 2016 Los Angeles Mommy and Baby (LAMB) follow-up study, 35% of women with normal weight BMI prepregnancy had transitioned to overweight or obesity by 2 years after giving birth [5]. Postpartum weight retention varies, and although many women return to their prepregnancy weight by 1 year post partum, a substantial proportion retain substantial amounts of weight [2,6]. In a cohort of women delivering their first child from Pennsylvania, 24% had retained 1-9 pounds (0.5-4 kg) and 24% had retained ≥10 pounds (4.5 kg) at 1 year post partum [6]. In the LAMB cohort, 35% had retained ≥10 pounds at 2 years post partum [5].

Although systematic reviews and meta-analyses have demonstrated the efficacy of lifestyle interventions targeting dietary intake and physical activity for weight loss during the postpartum period [7-10], interventions with numerous in-person sessions are not a good logistical match for the busy lives of many postpartum women [11-14]. Indeed, high attrition from treatment has plagued many postpartum weight loss intervention studies [7,8]. Remotely delivered lifestyle interventions can overcome some of the barriers to attending in-person meetings during the postpartum period (eg, work schedules, childcare, and transportation challenges) [11-14], challenges that have only increased during the COVID-19 pandemic [15]. In addition, remotely delivered lifestyle interventions may be more cost-effective to deliver, especially when accounting for participant costs [16]. Establishing noninferiority of remote versus in-person postpartum weight loss intervention models would advance the science by identifying a potentially more convenient and less costly model of care delivery.

Facebook may be an effective platform for remotely delivering evidence-based weight loss programming to postpartum women. Currently, 70% of US adults aged 18-29 years and 77% of adults aged 30-49 years use Facebook [17], with higher rates of use among mothers (87%) and women aged 18-39 years (84%) [18]. Many mothers turn to Facebook for support and information about parenting issues [19,20], and 80% of parents who use Facebook engage on the platform daily [18]. Using this popular commercial social media platform for intervention delivery allows us to leverage women’s daily routines to engage them in behavior change.

Lifestyle interventions that deliver at least some content via Facebook are efficacious for adults generally [21], and pilot studies conducted by our team and others have demonstrated feasibility and acceptability of leveraging Facebook for lifestyle intervention delivery among postpartum women specifically [22-25]. However, our approach is to deliver all the didactic intervention content via Facebook, whereas others have leveraged Facebook along with other treatment modalities (eg, telephone or in-person counseling sessions, text messaging, or an in-person orientation meeting) [23-25]. We previously developed a postpartum weight loss intervention [22] by adapting the Diabetes Prevention Program (DPP) lifestyle intervention [26] to address the needs of postpartum women and for delivery by a trained weight loss counselor via a private Facebook group [27]. In our earlier work, we conducted a 1-arm pilot study of our intervention with 19 postpartum women with overweight or obesity (ie, BMI ≥25 kg/m^2^ but <45 kg/m^2^) [22]. We were able to retain participants (95% retention) and keep them engaged over the 12-week intervention period [22]. The majority of participants said they would be likely or very likely to participate again if they had another baby, more than 80% would recommend the program to a postpartum friend, and participants lost an average of 4.8% of their baseline weight [22]. Although the results of this 1-arm pilot study are promising, this study did not provide information on the feasibility of recruitment of women able and willing to be randomized to either the Facebook or in-person intervention nor information about sustained participation in the Facebook-delivered intervention beyond 12 weeks. We conducted a randomized pilot trial to answer these feasibility questions (ie, feasibility of recruitment under conditions of randomization, sustained participation through the entire 6-month intervention, and retention at 6- and 12-month assessments) before conducting a large-scale trial to evaluate whether delivery via Facebook groups is noninferior to delivery via in-person group meetings.

### Objective

This study aimed to conduct a randomized feasibility pilot trial of a 6-month postpartum weight loss intervention delivered via Facebook versus in-person groups with postpartum women with overweight or obesity. We examined the feasibility of recruitment, sustained participation, contamination, retention, and assessment procedures in both conditions. We also described intervention acceptability. We described percent weight loss at 6 and 12 months in both treatment groups as an exploratory outcome.

## Methods

### Study Design

We conducted a randomized feasibility pilot trial to compare the delivery of a 6-month postpartum weight loss intervention via Facebook versus in-person groups among women with overweight or obesity. The design of this trial has been described in detail elsewhere [28].

### Ethics Approval

The University of Connecticut Institutional Review Board approved this study (protocol #H17-206). The trial was registered on clinicaltrials.gov (NCT03700736).

### Recruitment and Eligibility

Participants were recruited in 2 waves starting in August 2018 through October 2019. We recruited women from the Hartford, Connecticut area community by posting recruitment messages on Facebook, Instagram, Twitter, ResearchMatch [29], Craigslist, and University of Connecticut and UConn Health employee email digests, and by posting study flyers in the community. Additional details on recruitment are described elsewhere [28]. Research staff conducted eligibility screenings of interested individuals via phone.

Inclusion criteria were being aged ≥18 years; being at least 8 weeks but <12 months post partum at time of enrollment; having a BMI ≥25 kg/m^2^ per measured height and weight at baseline; either owning a scale or being willing to be provided one if needed; being comfortable reading and writing in English; owning an Android or iPhone smartphone; being an active Facebook user defined as accessing Facebook daily and posting or commenting at least weekly over the past 4 weeks; having clearance from their primary care provider or obstetrician or gynecologist; being willing to participate in either treatment condition (Facebook or in-person); being available to attend in-person meetings over the 6-month study period in Hartford, Connecticut; taking <45 minutes to travel to intervention meetings; and being willing and able to provide informed consent.

Women were excluded if they met any of the following criteria: currently pregnant; plan to become pregnant during the study period; current participation in an in-person or web-based clinical weight loss program; diagnosed with type 1 or type 2 diabetes as self-reported or reported by their health care provider; medical conditions or medications affecting weight; incapable of walking a quarter mile unaided without stopping; pain that prevents engagement in exercise; previous bariatric surgery; planned surgery during the study period; plans to move out of the area during the study period; high depressive symptoms or suicidal ideation (a score of ≥12 or positive on question #10 on the Edinburgh Postnatal Depression Scale [EPDS] [30]); positive screen for binge eating disorder [31]; failure to complete any baseline procedures (eg, baseline survey, orientation webinar, or prerandomization survey); or University of Connecticut student or employee supervised or taught by study investigators.

Eligible participants were all biologically female owing to the inclusion criteria of having given birth; we did not ask participants their gender identity. Although not all persons who become pregnant identify as women [32] as recruitment materials included the phase, “we are recruiting women who had a baby in the past year,” it is likely that all participants identified as women, and we refer to participants in this study as “women” or “mothers.”

### Assessments

Participants completed assessments at baseline, 6 months (postintervention), and 12 months, and filled out brief weekly surveys during the intervention period, as described in detail elsewhere [28]. Participants were provided gift cards to thank them for completing study assessments at baseline (US $20), 6 months (US $40), and 12 months (US $40). For wave 1, the intervention occurred from February to August 2019, with follow-up assessments in August 2019 and February 2020. For wave 2, the intervention occurred from October 2019 to April 2020, with follow-up assessments in April 2020 and October 2020.

At baseline, participants completed an in-person study visit that included providing informed consent, height and weight measurement, and screenings for elevated depressive symptoms and binge eating disorder. Study staff also provided instructions for downloading and using the MyFitnessPal app and instructions for using the battery settings to report Facebook app use (iPhone users) or a free app to track time on Facebook (Android users). Following this visit, participants completed a 30-minute web-based survey that included demographic and clinical characteristics (including prepregnancy weight for calculation of postpartum weight retention at baseline) and other baseline measures. Next, research staff contacted participants’ primary care provider or obstetrician or gynecologist for medical clearance. After completing their baseline visit and survey, participants completed a 60-minute webinar with other participants to orient them to the scientific process, review study procedures, and discuss the barriers and advantages of each study condition [33]. Following the orientation webinar but before randomization, participants completed a 5-minute web-based survey composed of a randomization agreement, report of app-tracked time on Facebook over the past 7 days, and their Facebook use habits [34]. Weekly during the intervention period, participants in both treatment conditions reported their weight, past 7-day app-tracked time on Facebook, and Facebook use habits [34] via a brief, 5-minute web-based survey.

At the end of the 6-month intervention, participants attended a focus group with other members of their weight loss group to provide qualitative feedback on their experiences in the study. The focus groups started out by asking general questions about participants’ experiences in the intervention (eg, “Overall, what do you think of this program?”, “What about this program did you find most helpful?”, and “How could we improve this weight loss program?”), transitioned to asking questions specific to each treatment modality (eg, “What influenced whether you commented on a post or comment?” in the Facebook condition and “How difficult was it for you to attend the sessions?” in the in-person condition), and finally prompted for any additional feedback (ie, “Do you have any other feedback about this program?”). Participants also completed a 30- to 45-minute web-based survey that included questions about contamination, acceptability, depressive symptoms (EPDS [30]), quality of life (PROMIS-Preference [PROPr] [35,36]), Facebook use habits including time spent on Facebook [17,34], and incident pregnancies. Research staff measured participants’ weight at the focus group visit. Participants who could not attend the focus group completed an individual interview and weight measurement at an individual visit. For wave 2, the 6-month focus groups were conducted via video conferencing software owing to the COVID-19 pandemic, and participants self-reported their current weight on the survey.

At 12 months, participants in wave 1 completed an in-person visit to measure weight and completed a 30-minute web-based survey that included measures of depressive symptoms (EPDS [30]), quality of life (PROPr [35,36]), Facebook use habits including time spent on Facebook [17,34], and incident pregnancies. Owing to the COVID-19 pandemic, participants in wave 2 did not attend in-person follow-up visits; follow-up weights were self-reported in the follow-up surveys.

### Randomization

Eligible participants who completed all screening and baseline procedures were randomized 1:1 to the Facebook and in-person conditions in randomly permuted blocks of size 4 and 6. Randomization was stratified by months post partum at enrollment (8 weeks to <6 months vs 6-12 months) and type of smartphone (iPhone vs Android). We stratified randomization by months post partum because weight change varies across the postpartum period in the absence of formal intervention [37,38]. We stratified randomization by smartphone type to balance any differences related to methods for measuring time spent on Facebook, as the procedures for collecting these data differed by phone operating system.

### Treatment Conditions

Participants in both treatment conditions received a 6-month weight loss intervention based on the DPP lifestyle intervention [26]. As described elsewhere [28], we adapted the intervention content to meet the needs and challenges of the postpartum period [11,39-42]. In the materials for both the in-person and Facebook-delivered interventions (eg, participant handouts and Facebook posts), we included stock images of women with larger bodies with a variety of skin tones, racial or ethnic phenotypes, and family configurations. Weight loss counselors had backgrounds in nutrition and dietetics and completed the National DPP training and training by a licensed clinical psychologist with extensive experience using the DPP in our specific intervention protocols [22,43,44]. The weight loss counselor for wave 1 identified as non-Hispanic White, and the weight loss counselor for wave 2 identified as Hispanic. The intervention goals were 5% to 10% weight loss and increasing physical activity to 150 minutes per week of moderate intensity physical activity. Calorie and physical activity goals were set to facilitate weekly weight loss of 1 to 2 pounds. For women who reported breastfeeding at baseline, initial calorie goals accounted for lactation [45], and calorie goals were adjusted during the intervention, as participants reported changes in breastfeeding. Participants were encouraged to use the free MyFitnessPal app to track their diet, exercise, and weight, and weight loss counselors emailed or messaged participants’ feedback on diet and activity records weekly or every 2 weeks (corresponding to the frequency of meetings in the in-person condition). Participants were withdrawn from the intervention if they reported becoming pregnant to the weight loss counselor or study staff. The 2 treatment conditions received the same intervention content; the difference between conditions was the delivery modality: in-person groups versus Facebook groups.

In the in-person condition, the weight loss counselor facilitated 90-minute group discussions, which were held weekly for the first 15 weeks and every other week during weeks 16-25, for a total of 20 meetings. The intervention materials were provided via paper handouts. Participants were reimbursed up to US $5 for parking or bus fare for each intervention meeting attended. Owing to the COVID-19 pandemic, the last 2 meetings of wave 2 were conducted via synchronous videoconferencing software.

In the Facebook condition, the weight loss counselor facilitated discussion about weekly topics via posts and comments in a private (“secret”) Facebook group [46]. The counselor posted 2 posts per day during weeks 1-15 and 1 post per day during weeks 16-25, corresponding to the intensity of contact in the in-person condition. We used the Facebook post scheduling tool to schedule daily intervention posts from the weight loss counselor’s account. We developed posts covering the intervention content of each module of the DPP lifestyle intervention based on our previous work with postpartum women [22] and adults generally [43,44,47]. Posts provided information and resources related to the topic of the week or asked participants to share their thoughts, experiences, or challenges related to the topic of the week; set goals (Mondays); report their progress toward these goals (Sundays); or report their weekly weight change (Fridays). Additional logistic details about the Facebook group and sample intervention posts are described elsewhere [28].

Participation in both interventions was monitored by the research team. The weight loss counselor recorded attendance at in-person intervention meetings. Research staff reviewed the Facebook group and recorded the date of each participant’s latest post or reply (each Monday during weeks 1-15 and every other Monday during weeks 16-25, corresponding to the frequency of in-person intervention meetings). The weight loss counselor emailed participants who did not participate (ie, did not attend in-person meetings or did not post or reply in the Facebook group) in a given week or 2-week period to encourage them to participate during the following week. After 2 consecutive weeks of no participation, the weight loss counselor called the participant, and after 3 consecutive weeks, the research coordinator called the participant. After 4 consecutive weeks without participation, the weight loss counselor sent a final email encouraging participation.

### Measures

#### Primary Outcomes: Feasibility

The feasibility outcomes were recruitment, retention, sustained participation, contamination, and feasibility of the assessment procedures. We also report participant feedback regarding the acceptability of the interventions.

#### Recruitment

We tracked participants through eligibility screening and study procedures and calculated recruitment rates from the number of individuals contacted, screened, consented, and randomized, overall and by recruitment source. We recorded the reasons for ineligibility and nonparticipation, including unwillingness to be randomized to either the Facebook or in-person condition.

#### Retention

We calculated retention as the proportion of participants who completed the 6- and 12-month follow-up assessments (ie, provided weight or completed the follow-up survey) in each condition.

#### Sustained Participation

We assessed sustained participation in the intervention (ie, treatment retention). For the in-person condition, the weight loss counselor recorded attendance at each intervention meeting, and sustained participation was calculated as the last intervention session attended. In the Facebook condition, treatment modules were spread over 1 week (weeks 1-15) or 2 weeks (weeks 16-25) to correspond to the frequency of intervention meetings in the in-person condition. Thus, we assessed participation in each treatment module. We captured engagement data from Facebook using Grytics tools (Grytics, Inc) and then manually abstracted identifiers (eg, participants’ Facebook usernames), reactions to posts and comments (including who reacted and what the reaction was), and poll responses (who voted for each option). A second member of the research team reabstracted a random 10% sample of threads (ie, a post plus any associated replies; 99.7% agreement across abstracted data points) to confirm the accuracy of abstraction. We calculated sustained participation as the latest treatment module participated in based on the latest post, reply (comments on posts and comments in replies to comments), poll vote (based on the date of the post that included the poll), or reaction (based on the date of the post or reply reacted to) in the Facebook group. We secondarily calculated whether participants participated in the last (20th) intervention module, and overall participation as the number of intervention modules participated in, and whether they participated in 0 intervention modules, ≥16 (ie, ≥80%), or 20 (ie, 100%). We additionally calculated the total number of posts, replies, polls voted in, and reactions by participants in the Facebook condition.

#### Contamination

To assess contamination, the 6-month follow-up survey included questions about participation in other weight loss programs (web-based or in person); whether participants sought weight loss support on Facebook or other web-based social networks [48]; and if so, to what extent and reasons they sought this support. One participant in the Facebook group reported during week 13 of the intervention that she had started a 21-week Beachbody program. Although she did not report this in her 6-month survey, we counted this as concurrent use of another weight loss program.

#### Feasibility of Assessment Procedures

We developed data collection and participant tracking systems and procedures. We assessed the degree of missingness of measures to be included in a large-scale trial to assess intervention efficacy and cost-effectiveness. We created a tracking system in REDCap (Research Electronic Data Capture; Vanderbilt University [49]) for research staff to enter time spent on specific tasks (eg, leading in-person intervention meetings, counseling via the Facebook group, copying participant handouts) [50] that would be needed to implement each intervention in practice (ie, outside the research context) using methods developed by others [51-53]. At baseline, 6 months, and 12 months, the participants completed a quality-of-life measure (PROPr) [35,36].

#### Acceptability of the Interventions

At 6 months, participants also answered questions regarding intervention acceptability [22]. Participants were asked, “If you had another baby, how likely would you be to participate in this weight loss program again?” and “If you had a friend who recently had a baby, how likely would you be to recommend this program to her?” (response options: “very unlikely,” “unlikely,” “neutral,” “likely,” and “very likely”; dichotomized as likely or very likely vs not). Participants were asked how convenient it was for them to log into the private Facebook group daily (Facebook condition) or attend 90-minute group meetings each week (in-person condition; response options: “very convenient,” “convenient,” “neither convenient nor inconvenient,” “inconvenient,” or “very inconvenient”; dichotomized as convenient or very convenient vs not). Participants in both groups were asked whether they would find attending weekly in-person group meetings or interacting in a private Facebook group daily more convenient (response options: “Facebook much more convenient,” “Facebook more convenient,” “Facebook and in-person groups equally convenient,” “in-person groups more convenient,” and “in-person groups much more convenient”; dichotomized as Facebook much more or more convenient vs not). To help us understand factors influencing intervention participation, after answering acceptability questions, participants were asked: “Thinking about the times when you logged in but did not post or reply to any posts why did you choose not to?” (Facebook condition) or “Thinking about the times when you didn’t come to the in-person group meetings, what was the reason?” (in-person condition). Participants in each condition were provided with a list of possible reasons (see tables for response options) and were asked to select all the answers that applied to them.

#### Exploratory Outcome: Weight Change

At baseline, 6 months, and 12 months, the staff measured weight twice (Tanita C-110 scale), and we calculated the average of the 2 measurements. In wave 1, participants who were unwilling to attend an in-person follow-up visit were asked to self-report their current weight (Facebook: 0/14, 0% at 6 months and 4/14, 29% at 12 months; in-person: 3/15, 20% at 6 months and 5/15, 33% at 12 months). As both follow-up time points for wave 2 occurred during the COVID-19 pandemic (April and October 2020), we pivoted to remote assessments, and participants self-reported their weight at 6 months and 12 months. Thus, all follow-up weights in wave 2 were self-reported. We calculated absolute (lbs) and percent weight change from baseline to 6 months and baseline to 12 months and defined clinically significant weight loss as ≥5% [54,55]. For women who were pregnant at follow-up, we used self-reported prepregnancy weight to calculate weight change. We secondarily calculated weight change assuming no weight change for those missing follow-up weights (baseline observation carried forward).

### Power Calculation

The purpose of this pilot trial was to examine feasibility and to identify modifications required before conducting a large randomized controlled trial to assess efficacy for weight loss. As recommended [56,57], we based the sample size on necessities for examining feasibility. We decided a priori that retention of ≥80% would indicate feasibility and that a retention rate in either condition <60% would indicate a lack of feasibility. With the target sample of 72 participants (36 per condition), the lower limit of the 95% CI for the observed retention rate in either treatment condition should not be <60%.

### Statistical Analysis

We used REDCap [49] for participant tracking and participant surveys. Data management and analyses were conducted using SAS (version 9.4; SAS Institute, Inc). We described the feasibility outcomes and exploratory outcome of weight loss in both conditions. We compared retention rates with the a priori benchmark for feasibility. After transcribing the focus group and interview recordings, we conducted a thematic analysis of responses [58]. This analysis focused on participant feedback about their assigned intervention modality that might impact acceptability ratings. Specifically, 3 members of the research team reviewed the focus group and interview transcripts as well as notes from focus group facilitators (familiarization) to create an initial list of feedback themes (generation of initial codes), reread transcripts to identify additional passages (searching for themes), and then consolidated feedback into themes (reviewing and defining themes) [58]. The first author then summarized relevant participant feedback identified by the review team (producing the report) [58].

## Results

### Study Sample

**Figure 1:**
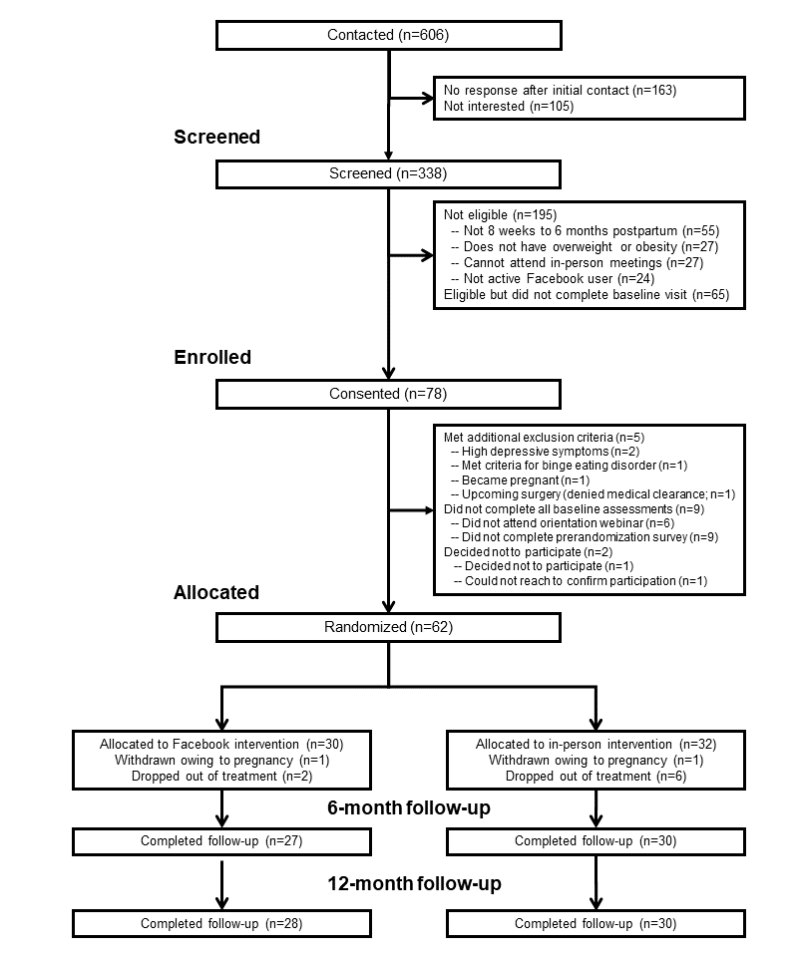
Participant recruitment and retention. Individuals excluded at eligibility screening and baseline assessment could be excluded for multiple reasons. Only the most common reasons for ineligibility are shown in the figure.

We screened 338 women, of whom 78 (23.1%) were eligible at screening and started baseline assessment procedures (Figure 1). We randomized 62 postpartum women. Two participants were withdrawn owing to pregnancy (1 per condition), and 8 dropped out of treatment (2 in the Facebook condition and 6 in the in-person condition; Figure 1). Overall retention was 92% (57/62) at 6 months and 94% (58/62) at 12 months.

Randomized participants (N=62) were on average aged 32.8 (SD 4.0) years and were a median of 6.1 (IQR 3.1-8.3) months post partum at enrollment (Table 1). In total, 60% (37/62) of participants had obesity at baseline, and median BMI was 31.7 (IQR 28.2-37.4) kg/m^2^. Average postpartum weight retention was 13.9 (SD 15.4) pounds. Most (43/62, 69%) of the participants were breastfeeding and 63% (38/62) had ≥2 children. Three-quarters (46/62, 74%) of participants were non-Hispanic White, 85% (53/62) had at least a bachelor’s degree, and 73% (40/62) were employed full-time. Additional characteristics of participants are provided in Table 1.

**Table 1:** Characteristics of postpartum women with overweight or obesity at study enrollment, overall, and by treatment condition.

			All randomized participants (n=62)		Facebook condition (n=30)		In-person condition (n=32)
**Smartphone type^a^, n (%)**							
	iPhone	45 (73)		21 (70)		24 (75)	
	Android	17 (27)		9 (30)		8 (25)	
**Months post partum^a^, median (IQR)**			6.1 (3.1-8.3)		5.8 (3.1-8.3)		6.1 (2.9-8.2)
	≥8 weeks but <6 months, n (%)	30 (48)		15 (50)		15 (47)	
	≥6 months but <12 months, n (%)	32 (52)		15 (50)		17 (53)	
Singleton gestation, n (%)			59 (95)		28 (93)		31 (97)
Breastfeeding, n (%)			43 (69)		23 (77)		20 (63)
>2 children in her household^b^, n (%)			38 (63)		21 (70)		17 (57)
**BMI (kg/m^2^), median (IQR)**			31.7 (28.2-37.4)		32.7 (28.0-36.6)		31.4 (28.3-37.6)
	Overweight, n (%)	25 (40)		12 (40)		13 (41)	
	Obesity, n (%)	37 (60)		18 (60)		19 (59)	
Postpartum weight retention (lbs), mean (SD)			13.9 (15.4)		14.6 (13.9)		13.1 (16.9)
Age (years), mean (SD)			32.8 (4.0)		33.3 (3.5)		32.3 (4.4)
**Race and ethnicity, n (%)**							
	Non-Hispanic White	46 (74)		22 (73)		24 (75)	
	Non-Hispanic Black	3 (5)		1 (3)		2 (6)	
	Hispanic or Latina	9 (15)		5 (17)		4 (13)	
	Non-Hispanic Asian	3 (5)		1 (3)		2 (6)	
	Non-Hispanic multiracial	1 (2)		1 (3)		0 (0)	
**Education, n (%)**							
	Less than bachelor’s degree	9 (15)		1 (3)		8 (25)	
	Bachelor’s degree or graduate courses	20 (32)		13 (43)		7 (22)	
	Graduate degree	33 (53)		16 (53)		17 (53)	
**Marital status, n (%)**							
	Married	55 (89)		28 (93)		27 (84)	
	Living with partner	5 (8)		2 (7)		3 (9)	
	Single	2 (3)		0 (0)		2 (6)	
**Employment status^b^, n (%)**							
	Employed full-time	40 (73)		19 (73)		21 (72)	
	Employed part-time	8 (15)		4 (15)		4 (14)	
	Stay-at-home mom (not employed)	7 (13)		3 (12)		4 (14)	
**Hard to pay for basics, n (%)**							
	Not at all hard	37 (60)		16 (53)		21 (66)	
	Somewhat hard	24 (39)		13 (43)		11 (34)	
	Very hard	1 (2)		1 (3)		0 (0)	

^a^Randomization was stratified by smartphone type and months post partum.

^b^n=7 participants missing information on employment (n=4 in the Facebook condition and 3 in the in-person condition), and n=2 participants missing information on number of children in her household (both in the in-person condition).

### Feasibility of Recruitment

Among individuals who were not interested in the study and therefore not screened for eligibility, 68.6% (72/105) explicitly reported a lack of interest or barriers related to the in-person condition as the reason (Table 2). In contrast, only 2.9% (3/105) were not interested in participating because they were not interested in the Facebook condition.

Among individuals determined to be ineligible at screening, 33.3% (65/195) were ineligible owing to reasons related to one or both treatment modalities, 18.5% (36/195) owing to reasons related to the in-person condition (ie, not available on the day or time of meetings, >45 minutes of travel to the intervention location, or plans to move out of the area in the next 12 months), 2.6% (5/195) owing to unwillingness to be randomized to either condition (all 5 preferred Facebook), and 12.3% (24/195) owing to their Facebook use habits (eg, no Facebook account, does not browse their feed at least daily, or does not post or reply at least weekly).

**Table 2:** Reasons^a^ potential participants were not interested in participating in the study (N=105).

			Values, n (%)
**Any reason related to in-person condition**			72 (69)
	Not interested in in-person condition	51 (49)	
	Day or time of meetings does not work	29 (28)	
	Intervention location too far to travel	19 (18)	
**Any reason related to Facebook condition**			3 (3)
	Not interested in Facebook condition	3 (3)	
**Reasons not explicitly related to either treatment modality**			24 (23)
	Does not have time to participate	24 (23)	
	Intervention will not start soon enough	0 (0)	

^a^Participants could provide multiple reasons for not being interested in participating in the study.

### Retention

Overall, retention was 92% (57/62) at 6 months and 94% (58/62) at 12 months. Retention in the Facebook condition was 90% (27/30) at 6 months and 93% (28/30) at 12 months. Retention in the in-person condition was 94% (30/32) at 6 months and 94% (30/32) at 12 months. Retention in both conditions at both time points exceeded our a priori benchmarks of 80%, and the lower limits for 95% CIs were ≥79% for all conditions at all time points, which was substantially higher than the a priori benchmark of 60%.

Because of the disruptions to study procedures caused by the COVID-19 pandemic, we also explored retention by wave. In wave 1, retention in the Facebook condition was 79% (11/14) at 6 months and 86% (12/14) at 12 months and 93% (14/16) at 6 months and 93% (14/15) at 12 months in the in-person condition. In wave 2, retention in the Facebook condition was 100% (16/16) at 6 months and 100% (16/16) at 12 months and 94% (16/17) at 6 months and 94% (16/17) at 12 months in the in-person condition.

### Sustained Participation and Engagement

Over the 6-month intervention, participants in the Facebook condition posted 159 original posts and 3318 replies and contributed 614 poll votes and 1996 reactions. Participants posted a median of 3 (IQR 1-7; range 0-28) original posts and a median of 88 (IQR 50-142; range 11-309) replies and contributed a median of 18.5 (IQR 11-29; range 3-59) poll votes and a median of 47 (IQR 24-94; range 7-208) reactions.

In total, 70% (21/30) of the participants in the Facebook condition posted, replied, voted in a poll, or reacted during the last 2 weeks of the intervention (ie, participated in the last intervention module), compared with 31% (10/32) of participants in the in-person condition who attended the final intervention meeting (Table 3). The latest treatment module participated in (of 20 modules) was median 20 (IQR 19-20) for participants in the Facebook condition and median 18 (IQR 2-20) for participants in the in-person condition (Table 3). In a sensitivity analysis that used a stricter definition of participation for participants in the Facebook condition—posting and replying only—63% (19/30) of participants participated in the last intervention module, and the latest treatment module participated in was median 20 (IQR 17-20). Participants posted or replied in a median of 18 (IQR 13-20) treatment modules; 67% (20/30) of participants engaged in ≥16 modules, and 43% (13/30) engaged in all 20 intervention modules.

In wave 2, the last 2 intervention meetings for the in-person condition were held via videoconference calls owing to the COVID-19 pandemic. In wave 1, a total of 33% (5/15) and 33% (5/15) of participants, respectively, attended the 19th and 20th intervention meeting. In wave 2, a total of 35% (6/17) and 29% (5/17) of participants, respectively, attended these last 2 meetings. In the postintervention focus groups, some participants mentioned liking not needing to travel or arrange childcare, but others mentioned “Zoom fatigue,” feeling less connected to other women over video versus in person, or having children in the background was distracting.

When participants in the Facebook condition were asked to select reasons they did not post or reply when they had logged into Facebook, the most common responses selected were not having anything to add to the conversation (22/26, 85%), preferring to lurk rather than actively engaging (13/26, 50%), and not wanting to be the only person posting (10/26, 38%; Table 4). In-person participants were similarly asked why they did not attend their in-person meetings. The most common response, endorsed by 93% (25/27) of participants, was the need to attend to other responsibilities that were more important (Table 4). Other common responses included motivation for weight loss declined (4/27, 15%) and forgetting about the meeting (4/27, 15%; Table 4).

**Table 3:** Sustained participation^a^ in the intervention, by treatment condition.

			Facebook condition (n=30)		In-person condition (n=32)
**Latest intervention module participated in, median (IQR)**			20 (19-20)		18 (2-20)
	Participated in the last intervention module, n (%)	21 (70)		10 (31)	
**Number of intervention modules participated in, median (IQR)**			19 (17-20)		10 (2-14)
	Participated in no intervention modules, n (%)	0 (0)		6 (19)	
	Participated in ≥16 (ie, ≥80%) intervention modules, n (%)	24 (80)		7 (22)	
	Participated in all 20 intervention modules, n (%)	13 (43)		0 (0)	

^a^Participation in a treatment module was attending the intervention meeting for participants in the in-person condition and posting, replying, voting in a poll, or reacting to a post or comment in the Facebook condition.

**Table 4:** Reasons participants did not post in the Facebook group or attend in-person intervention meetings, by condition.

		Value, n (%)
**Reasons participants in the Facebook condition did not post or reply when they logged into the Facebook group (N=26)**		
	I did not have anything to add to the conversation	22 (85)
	I generally prefer to “lurk”—meaning I like to read posts but not say anything	13 (50)
	It seemed like nobody in the group was posting so I did not want to be the only one	10 (38)
	The topic was not relevant to me	6 (23)
	I did not feel comfortable posting my ideas	4 (15)
	The topic was not interesting to me	4 (15)
	I feared how people would respond to what I would say (eg, I might get judged, ignored, or not supported)	3 (12)
	I did not feel like I was a part of the group	1 (4)
	I did not understand how to post	0 (0)
	I was concerned about privacy in the group	0 (0)
**Reasons participants in the in-person condition did not attend the group intervention meetings (N=27)**		
	I had to attend to other responsibilities that were more important	25 (93)
	My motivation to focus on weight loss declined	4 (15)
	I forgot we had group that day	4 (15)
	I had transportation issues	3 (11)
	It seemed like a lot of people weren’t coming so this reduced my motivation to be a part of the group	3 (11)
	The topics were not interesting or helpful to me	2 (7)
	I did not feel like I was a part of the group	2 (7)
	The topic was not relevant to me	1 (4)
	I did not feel comfortable in the group	1 (4)
	I was concerned about privacy in the group	0 (0)
	I feared how people would respond to me (eg, I might get judged, ignored, or not supported)	0 (0)
	N/A^a^ (I attended all groups)	0 (0)

^a^N/A: not applicable.

### Contamination

In total, 15% (4/26) of participants in the Facebook condition and 4% (1/28) of participants in the in-person condition reported that they had used other in-person or web-based weight loss programs during the intervention period. However, when asked for details about these other weight loss programs, only 2 participants reported a structured program (Weight Watchers, a 21-week Beachbody program). The other 3 participants reported activities that would support their weight loss efforts (eg, saw a nutritionist, used a meal plan from dietitian she has been working with for 4 years, and personal training challenge) but do not represent a structured weight loss program. In total, 35% (9/26) of participants in the Facebook condition and 57% (16/28) of participants in the in-person condition sought weight loss information or support on social media during the intervention period. All reports of contamination were to external resources; no participants reported access to the other study intervention.

### Feasibility of Assessment Procedures

Regarding patient-reported data needed to evaluate cost-effectiveness, we were able to obtain measured baseline weights on 100% (62/62) of participants in both conditions and measured or self-reported weights for 90% (27/30) and 93% (28/30) of participants in the Facebook condition at 6 and 12 months, respectively, and 94% (30/32) and 94% (30/32) of participants in the in-person condition, respectively. At baseline, 1 participant in each condition (2/62, 3%) did not complete the quality-of-life measure (PROPr) owing to a license agreement issue that delayed inclusion of another quality-of-life measure in the baseline survey. An additional 4 participants missed one of the PROPr items; at baseline, 87% (26/30) and 94% (30/32) of participants in the Facebook and in-person conditions, respectively, completed the quality-of-life measure in full. At 6 and 12 months, respectively, 87% (26/30) and 93% (28/32) of participants in the Facebook condition and 84% (27/30) and 91% (29/32) of participants in the in-person condition completed this measure in full.

### Acceptability

Half (13/26, 50%) of participants in the Facebook condition would be likely or very likely to participate again and 54% (14/26) would be likely or very likely to recommend this program to a friend (Table 5). In the in-person condition, 58% (15/26) of participants would participate again and 70% (19/27) would recommend this program to a friend (Table 5). In postintervention focus groups or interviews, participants in the Facebook condition shared that they appreciated the flexibility of being able to engage with the group anytime and from anywhere. However, participants also noted that a downside of this flexibility was a lack of accountability—that it was easy to put off responding to posts or setting goals because there was no set schedule for participation. Participants also noted that they felt it was hard to get to know other mothers and build a sense of community. In contrast, participants in the in-person condition shared that they really got to know other women in their group and felt a strong sense of community. They also felt that meeting in person—and being weighed at intervention meetings—kept them accountable, and having a set meeting time each week helped them prioritize their health. However, participants noted barriers including time to travel to meetings, variable parking availability, and the need to arrange childcare for older children. A few participants also mentioned feeling guilty leaving their children 1 evening per week.

**Table 5:** Intervention acceptability by treatment condition.

	Facebook condition (n=26), n (%)	In-person condition (n=27), n (%)
If you had another baby, how likely would you be to participate in this weight loss program again? (likely or very likely)^a^	13 (50)	15 (58)
If you had a friend who recently had a baby, how likely would you be to recommend this program to her? (likely or very likely)	14 (54)	19 (70)
How convenient was it for you to log into the private Facebook group each day/attend 90-minute group meetings each week? (convenient or very convenient)	25 (96)	2 (7)
Thinking about attending weekly in-person group meetings versus interacting in a private Facebook group daily, which would you find more convenient? (Facebook more convenient or Facebook much more convenient)^a^	23 (88)	23 (88)

^a^n=1 participant in the in-person condition missing information for this question.

Almost all participants (25/26, 96%) in the Facebook condition reported that it was convenient or very convenient for them to log into the private Facebook group each day (Table 5). In contrast, only 7% (2/27) of participants in the in-person condition said it was convenient or very convenient to attend 90-minute group meetings each week. Most of the participants (23/26, 88%) in each condition agreed that interacting in a private Facebook group daily would be more convenient than attending a weekly in-person group (Table 5). In postintervention focus groups, several participants suggested a hybrid approach. A few participants from the Facebook condition suggested adding video meetings to increase accountability and sense of community, whereas others suggested occasional in-person meetings (eg, to start the group or once a month). Participants from the in-person condition suggested adding a Facebook group for connection and support between in-person meetings.

### Weight Change (Exploratory Outcome)

At 6 months, participants in the Facebook condition had lost an average of 3.0% (SD 7.2%) of their baseline weight and participants in the in-person condition had lost an average of 5.4% (SD 6.8%; Table 6). Average percent weight loss at 12 months was 2.8% (SD 7.4%) in the Facebook condition and 4.8% (SD 7.6%) in the in-person condition (Table 6). In a sensitivity analysis using a baseline observation carried forward approach (ie, assuming no weight change) for participants missing follow-up weights (3 at 6 months and 2 at 12 months in the Facebook condition and 2 and 2 in the in-person condition), average percent weight loss at 6 months was 2.7% (SD 6.9%) in the Facebook condition and 5.0% (SD 6.7%) in the in-person condition, with 27% (8/30) and 50% (16/32), respectively, achieving ≥5% weight loss. At 12 months, average percent weight loss was 2.6% (SD 7.2%) in the Facebook condition and 4.5% (SD 7.5%) in the in-person condition, with 33% (10/30) and 47% (15/32), respectively, achieving 5% weight loss.

**Table 6:** Weight change at 6 and 12 months, by treatment condition.

		Facebook condition^a^	In-person condition^a^
**Weight change from baseline (lbs), mean (SD)**			
	6 months	−4.8 (13.8)	−10.0 (13.0)
	12 months	−5.1 (13.8)	−9.2 (15.3)
**Weight change from baseline (%), mean (SD)**			
	6 months	−3.0 (7.2)	−5.4 (6.8)
	12 months	−2.8 (7.4)	−4.8 (7.6)
**Lost ≥5% of baseline weight, n (%)**			
	6 months	8 (30)	16 (53)
	12 months	10 (36)	15 (50)

^a^At 6 months, weights were available for 27 participants in the Facebook condition and 30 participants in the in-person condition. At 12 months, weights were available for 28 participants in the Facebook condition and 30 participants in the in-person condition.

## Discussion

### Principal Findings

Feasibility trials provide an opportunity to pilot study procedures and measures in preparation for a large-scale efficacy trial. We assessed the feasibility of recruiting a sample of postpartum women willing and able to participate in a lifestyle intervention delivered either via Facebook or in-person groups. The in-person condition posed challenges to recruitment. Among individuals not interested in the study, 68.6% (72/105) were not interested in or could not attend in-person meetings, and 18.5% (36/195) of screened individuals were excluded because of reasons related to participating in the in-person condition. These findings are not unexpected, as the barriers to attending in-person treatment sessions documented in several previous studies [11-14,41,59-61] motivated the current line of research to develop a Facebook-delivered version of the intervention. The numbers of participants contacted, screened, eligible, and enrolled will inform the timeline for subsequent efficacy testing.

Overall, retention was 92% at 6 months and 94% at 12 months. Although based on the small numbers (approximately 15 participants per condition per wave), it appears that retention may have been higher in the Facebook condition in wave 2 when in-person assessments were not required. In wave 1, retention at 6 and 12 months was 79% and 86% versus 100% and 100%, respectively, in wave 2. For comparison, retention in the in-person condition was 93% and 93% in wave 1 and 94% and 94% in wave 2 at 6 and 12 months, respectively. It may be that women who were used to connecting with the weight loss counselor and their group remotely perceived attending an in-person visit as more burdensome. The only data collected at follow-up that required an in-person visit were weight. Providing participants digital scales in future trials would allow all follow-up assessments to be conducted remotely [62], which may increase completion of follow-up measures by reducing participant burden. Remote assessments would also allow for national recruitment, thus widening the participant pool.

Average weight losses of 3% in the Facebook condition and 5% in the in-person condition are promising, and it appears that women in the in-person condition lost more weight on average than those in the Facebook condition. Future research should explore the mechanisms through which delivering lifestyle interventions in person versus via digital platforms influences weight loss (eg, through increased accountability, stronger connections with the weight loss counselor or group members, and greater impact on participants’ motivation to engage in behavior change). However, the weight loss in this trial should be interpreted cautiously for 2 reasons. First, participants in wave 2 self-reported their weight at 6 months and 12 months owing to the COVID-19 pandemic-related disruptions in in-person research assessments. Although weights self-reported as part of a digital lifestyle intervention tend to be accurate [63,64], weights measured with home scales that differ by a few pounds from the study scale can bias estimates of weight change, especially when baseline weights are measured by study staff. Second, the COVID-19 pandemic and particularly the early-pandemic school and childcare shutdowns had immeasurable impact on women’s lives and their motivation and ability to make and sustain behavioral changes [65,66]. A study on the impact of the COVID-19 pandemic on current research participants’ ability and desire to engage in research found that among those currently enrolled in a group-based behavioral intervention, 52% reported that the pandemic had impacted their ability to adhere to behavioral recommendations a little bit or moderately and 22% reported that the pandemic had impacted their behavior quite a bit or extremely [66]. Indeed, multiple participants in wave 2 reported at their 12-month follow-up assessments that they had regained a substantial amount of weight owing to disruptions and stress related to the pandemic, whereas others said that they lost additional weight owing to changes in their lifestyle (eg, sharp decline in eating out and more exercise). As only a single wave of participants completed the study before the pandemic and a single wave had their experience disrupted by the COVID-19 pandemic, we did not have a sufficiently large sample size to examine the impact of the pandemic on participant experiences or outcomes.

Whether participants stay engaged in treatment influences treatment receipt and, thus, efficacy. Digital health interventions have long been plagued by high dropout rates [67], and systematic reviews of postpartum weight loss interventions have highlighted attrition from treatment as a common challenge [7,8]. In our previous 1-arm pilot of the Facebook-delivered intervention, 63% of participants participated in the last week of the intervention [22]; however, that preliminary pilot study did not provide information on whether participants would stay engaged in treatment for the full 6-month intervention. This feasibility pilot trial examined sustained treatment through the full 6-month intervention period in both the Facebook-delivered and in-person versions of the intervention and found that 70% (21/30) of women in the Facebook condition engaged in the Facebook group during the last intervention module (last 2 weeks of the intervention) and engaged during a median of 19 (IQR 17-20) of the 20 intervention modules. In contrast, only 31% (10/32) of participants in the in-person condition attended their last intervention meeting. Women only attended a median of 10 (IQR 2-14) of the 20 intervention meetings, and 19% (6/32) did not attend a single intervention meeting. To be sure, participating in Facebook requires much less effort than attending a group visit. Indeed, when asked to select from a list of reasons participants did not come to the meetings, 93% (25/27) indicated that they had to attend to other responsibilities, including being sick, caring for an ill child, work, or caring for older children while their spouse worked, emphasizing the challenges of in-person meetings for mothers. Although we defined sustained participation in terms of the latest intervention module participated in (ie, in-person meeting attended or latest intervention module with visible engagement in the Facebook group), this definition would allow participants with large gaps in participation to pop back into the group at the end of treatment and be counted as having sustained participation. Thus, we recommend not only examining the time to last participation but also the number of treatment sessions or modules to provide a more comprehensive picture of treatment receipt.

In addition to examining sustained participation, we also described engagement in the Facebook-delivered intervention. Over the 6-month intervention, participants in the Facebook condition posted a median of 3 (IQR 1-7; range 0-28) original posts and a median of 88 (IQR 50-142; range 11-309) replies. Previous studies delivered a weight loss intervention based on the DPP lifestyle intervention entirely via a commercial social media platform for 12 weeks; thus, we additionally calculated engagement for the first 12 weeks of this study. Participants posted a median of 3 (IQR 1-5) original posts and a median of 65 (IQR 40-93) replies. Engagement over the first 12 weeks was higher in this study than in our previous 1-arm pilot study of a 12-week version of the Facebook-delivered intervention in which participants contributed a median of 2 (IQR 1-3) original posts and a median of 24 (IQR 15-31) replies [22]. We purposefully revised our intervention posts in response to engagement findings and participant feedback. It may be that the updated intervention posts were more effective at eliciting engagement from participants. Engagement in this study also appeared to be higher than engagement in other Facebook-delivered lifestyle interventions. In our previous research of a 12-week Facebook-delivered weight loss intervention with adults with obesity generally, participants shared a median of 37 (IQR 16-76) original posts or replies [47], less than the median of 68 (IQR 40-93) posts and replies shared during the first 12 weeks in this study (median 92.5, IQR 53-153 over 6 months). Women in this study also appeared to engage more than participants randomized to the comparison condition of a pilot study testing an app to track dietary lapses (median 0 posts, IQR 0-1; median 29.5 replies, IQR 17-61) [68]. The median number of replies over the first 12 weeks of the intervention posted by women in this study was also about twice the median number of replies of other Facebook-delivered weight loss interventions among adults [69,70] and higher than the average engagement in pilot trials of postpartum weight loss interventions that delivered some or all content via Facebook [24].

Despite higher engagement than other Facebook-delivered weight loss interventions, when asked to select reasons why they did not post or reply in the group, 38% (10/26) of participants endorsed “It seemed like nobody in the group was posting so I didn’t want to be the only one.” This feedback may be related to participants being hesitant to be the first one to respond to an intervention post, as we have heard from participants in previous studies [22]. This feedback may also be partially explained by when these questions were asked. The weight loss counselor posted in the Facebook group twice per day during weeks 1-15 of the intervention and then once a day in weeks 16-25; therefore, participants’ reports of their experiences at the 6-month follow-up assessment may be biased toward their more recent experiences in the group compared with the activity in the group over the full 6-month intervention. Future studies could explore whether maintaining a posting schedule of twice daily results in greater participant engagement in the latter weeks of the intervention. Future studies could also experiment with ways to encourage participants to start conversation threads, so that the volume of conversation in the group is less dependent on the weight loss counselor’s posts.

Participants also indicated that they did not post or reply in the group because they did not have anything to add to the conversation (22/26, 85%) or the topic was not relevant (6/26, 23%) or interesting (4/26, 15%) to them. In addition, 50% (13/26) of participants noted that they generally preferred lurking over visibly engaging. In one of our previous social media-delivered weight loss interventions, participants who had not used the social media platform before enrolling in the study engaged less in the intervention [43]. In our 1-arm pilot of the Facebook-delivered postpartum weight loss intervention, participants reported in postintervention focus groups that their typical social media habits influenced their level of engagement in the intervention [22]. Thus, we limited enrollment in this study to women who reported posting or replying on Facebook at least weekly. Future research should explore how to convert lurkers into posters as a strategy to boost engagement in the group. Future research could also explore the potential benefits participants experience from reading conversation threads without visibly engaging in them [71].

Another option to increase engagement in digital weight loss groups may be to increase the number of women receiving a postpartum weight loss intervention via a private Facebook group. Pagoto et al [69] recently conducted a proof-of-concept pilot study comparing engagement in a Facebook-delivered lifestyle intervention in which group membership was allowed to grow with engagement in a group in which membership was static. Although total engagement (original posts, replies, poll votes, and reactions) did not differ among the 40 participants initially randomized to the open enrollment group compared with the 40 participants randomized to the closed group, total engagement was higher among all 94 participants in the open enrollment group, and the total volume of engagement contributed by participants and weight loss counselors was associated with participants’ weight loss [69]. As Facebook’s algorithms prioritize groups with more activity [72], larger groups with more participant posts and replies may be prioritized in women’s Facebook feeds, thus increasing the opportunity to engage and subsequently better treatment receipt.

Although engagement in the Facebook condition in this study was higher than that in our previous work with postpartum women or previous studies with adults with obesity, participants appeared to lose less weight. Average weight loss of 3% over 6 months among participants in the Facebook condition was lower than the average weight loss of 4.8% observed in our previous 1-arm pilot study of a 12-week version of the Facebook-delivered intervention [22]. Weight loss among participants in the Facebook condition in this study was similar to the average weight loss achieved in studies of adults generally over shorter periods (ie, 12 or 16 weeks) [47,69,70]. Differences in the samples, including the requirement of being willing to participate in either an in-person or digital intervention and enrollment of women earlier in the postpartum period (average 3.4 months post partum in the 1-arm pilot vs median 6.1 months in this study), may have also contributed to differences in weight loss. Although our weight loss findings should be interpreted with caution, these findings suggest that a deeper investigation into what types of engagement are associated with weight loss during the postpartum period is warranted. Not all utterances in a Facebook weight loss group are associated with weight loss [47], and additional research is needed on how best to engage participants in interactions that lead to successful behavior changes and subsequently weight loss [73].

Taken together, our findings related to the feasibility of recruitment under conditions of randomization to an in-person or Facebook condition and our findings related to participation in in-person intervention meetings indicate that an in-person weight loss program with numerous visits has limited feasibility for many postpartum persons. Further research is needed to develop and test efficacious postpartum weight loss interventions that work with postpartum persons’ busy lives. Synchronous video meetings may be an option to foster group cohesion and accountability while overcoming the logistic challenges of in-person meetings. Telehealth or video visits offer the opportunity to connect individuals and groups face-to-face while retaining many of the benefits of in-person interactions. The national telehealth landscape has changed significantly since the initiation of this study. The COVID-19 pandemic has inspired a rapid uptake of telehealth in clinical settings [74,75], including obstetric care [76]. Indeed, in this study, the COVID-19 pandemic necessitated a shift of modality for the last 2 intervention meetings for wave 2 from in-person to videoconferencing. Attendance at these meetings was very similar to attendance at the analogous meetings in wave 1, and women liked not having to travel or arrange childcare. However, some participants mentioned “Zoom fatigue” [77], not surprisingly, as this feedback was provided in April 2020, approximately a month after the COVID-19–related shutdowns. As more and more activities resume in person, “Zoom fatigue” is likely to lessen. Other weight loss trials that transitioned from in-person to video meetings also found this modality acceptable, and participants lost weight [78,79]. Weight loss interventions based on the DPP lifestyle intervention have been successfully delivered via video meetings [80]. Women from a wider geographic range can be enrolled without travel constraints to attend in-person intervention meetings. Video meetings might also alleviate barriers to participation related to childcare, as women could participate in groups while their children sleep, engage in other activities at their home, or participate during lunch or another break during their workday. Another option may be a hybrid approach [81], such as an intervention delivered primarily remotely with a few in-person meetings, an approach that has been shown to be effective in low-income postpartum women [82]. Future research could explore the acceptability and efficacy of delivering a postpartum weight loss intervention via synchronous video group meetings, either in place of in-person meetings or in addition to an intervention delivered primarily digitally.

This feasibility trial also provided an opportunity to pilot and reflect on how contamination was measured. When we designed this study, we defined contamination in 2 ways: participation in other digital or in-person weight loss programs and seeking weight loss information or support on Facebook or other digital social networks. As 3 of the 5 participants who responded affirmatively to a question about concurrent participation in a structured weight loss program reported activities that did not meet our definition of a structured program, in future studies, we will revise the survey question and also have staff call participants to obtain additional details about professional assistance with weight loss outside the study intervention. As the study progressed, we realized that defining seeking weight loss information or support on social media as contamination did not match our study protocols, as we directed women in both conditions to a study Pinterest account where we had compiled online resources helpful for their weight loss journeys (eg, low-calorie recipes, workout videos, and MyFitnessPal tutorial videos) and encouraged women on Facebook to create a healthy Facebook feed by following Pages by public health organizations (eg, AHALiveHealthy and EatRightNutrition). In future studies, we will focus on contamination tracking of participation in structured weight loss programs or weight loss-specific Facebook groups.

### Limitations

An additional limitation of this study is its limited racial, ethnic, and socioeconomic diversity. Our sample was more highly educated than US women giving birth overall (53/62, 85% with a bachelor’s or higher education vs 34% nationally), more likely to be non-Hispanic White (46/62, 74% vs 51% nationally), and less likely to be unmarried (7/62, 11% vs 40% nationally) [83]. Many behavioral weight loss trials struggle to recruit racially or ethnically diverse samples [84,85]. In future studies, we will use strategies to diversify the participant pool. Targeted recruitment advertisements and strategic placement of such materials can facilitate the recruitment of an ethnically and economically diverse sample into weight loss trials [86]. Effective strategies to recruit low-income and racially or ethnically diverse postpartum women and parents of young children into behavioral trials include working with community partners (eg, Women, Infants, & Children Nutrition Program [WIC]), hiring culturally representative and sensitive research staff, and having multiple contacts with potential participants [87,88]. In future studies in this line of research, we will ask interested individuals to provide some demographic information (eg, Hispanic ethnicity, race, education, and participation in WIC or Supplemental Nutrition Assistance Program) earlier in the eligibility screening process so that we can monitor participant yield and characteristics from different recruitment approaches and refine our strategies to yield a more racially, ethnically, and socioeconomically diverse sample.

### Conclusions

Delivering a lifestyle intervention to postpartum women via both in-person and Facebook groups was feasible and acceptable and resulted in weight loss. However, barriers to attending in-person meetings hampered recruitment efforts and attendance at in-person intervention meetings. Although women found the Facebook group convenient and stayed engaged in the group, weight loss appeared lower than that with in-person delivery. Research is needed to further develop care models for postpartum weight loss that balance accessibility with efficacy.

## Abbreviations

DPP: Diabetes Prevention Program
EPDS: Edinburgh Postnatal Depression Scale
LAMB: Los Angeles Mommy and Baby
PROPr: PROMIS-Preference
REDCap: Research Electronic Data Capture
WIC: Women, Infants, & Children Nutrition Program

## References

[1] Endres LK, Straub H, McKinney C, Plunkett B, Minkovitz CS, Schetter CD, Ramey S, Wang C, Hobel C, Raju T, Shalowitz MU, Community Child Health Network of the Eunice Kennedy Shriver National Institute of Child Health and Human Development (2015). Postpartum weight retention risk factors and relationship to obesity at 1 year. Obstet Gynecol.

[2] Gore SA, Brown DM, West DS (2003). The role of postpartum weight retention in obesity among women: a review of the evidence. Ann Behav Med.

[3] Gunderson EP, Murtaugh MA, Lewis CE, Quesenberry CP, West DS, Sidney S (2004). Excess gains in weight and waist circumference associated with childbearing: the Coronary Artery Risk Development in Young Adults Study (CARDIA). Int J Obes Relat Metab Disord.

[4] Abrams B, Heggeseth B, Rehkopf D, Davis E (2013). Parity and body mass index in US women: a prospective 25-year study. Obesity (Silver Spring).

[5] Archuleta J, Chao SM (2021). Maternal characteristics that impact postpartum weight retention: results from the 2016 Los Angeles Mommy and Baby (LAMB) follow-up study. Matern Child Health J.

[6] Legro NR, Lehman EB, Kjerulff KH (2020). Mode of first delivery and postpartum weight retention at 1 year. Obes Res Clin Pract.

[7] Amorim Adegboye AR, Linne YM (2013). Diet or exercise, or both, for weight reduction in women after childbirth. Cochrane Database Syst Rev.

[8] Dodd JM, Deussen AR, O'Brien CM, Schoenaker DA, Poprzeczny A, Gordon A, Phelan S (2018). Targeting the postpartum period to promote weight loss: a systematic review and meta-analysis. Nutr Rev.

[9] Lim S, Liang X, Hill B, Teede H, Moran LJ, O'Reilly S (2019). A systematic review and meta-analysis of intervention characteristics in postpartum weight management using the TIDieR framework: a summary of evidence to inform implementation. Obes Rev.

[10] Sherifali D, Nerenberg KA, Wilson S, Semeniuk K, Ali MU, Redman LM, Adamo KB (2017). The effectiveness of eHealth technologies on weight management in pregnant and postpartum women: systematic review and meta-analysis. J Med Internet Res.

[11] Carter-Edwards L, Østbye T, Bastian LA, Yarnall KS, Krause KM, Simmons TJ (2009). Barriers to adopting a healthy lifestyle: insight from postpartum women. BMC Res Notes.

[12] Montgomery KS, Bushee TD, Phillips JD, Kirkpatrick T, Catledge C, Braveboy K, O'Rourke C, Patel N, Prophet M, Cooper A, Mosley L, Parker C, Douglas GM (2011). Women's challenges with postpartum weight loss. Matern Child Health J.

[13] Graham M, Uesugi K, Olson C (2016). Barriers to weight-related health behaviours: a qualitative comparison of the socioecological conditions between pregnant and post-partum low-income women. Matern Child Nutr.

[14] Ryan RA, Lappen H, Bihuniak JD (2022). Barriers and facilitators to healthy eating and physical activity postpartum: a qualitative systematic review. J Acad Nutr Diet.

[15] Schaeffer K (2022). Working moms in the U.S. have faced challenges on multiple fronts during the pandemic. Pew Research Center.

[16] Krukowski RA, Tilford JM, Harvey-Berino J, West DS (2011). Comparing behavioral weight loss modalities: incremental cost-effectiveness of an internet-based versus an in-person condition. Obesity (Silver Spring).

[17] Auxier B, Anderson M (2021). Social media use in 2021. Pew Research Center.

[18] Waring ME, Blackman Carr LT, Heersping GE (2023). Social media use among parents and women of childbearing age in the US. Prev Chronic Dis.

[19] Duggan M, Lenhart A, Lampe C, Ellison NB (2015). Parents and social media: mothers are especially likely to give and receive support on social media. Pew Research Center.

[20] Holtz B, Smock A, Reyes-Gastelum D (2015). Connected motherhood: social support for moms and moms-to-be on Facebook. Telemed J E Health.

[21] An R, Ji M, Zhang S (2017). Effectiveness of social media-based interventions on weight-related behaviors and body weight status: review and meta-analysis. Am J Health Behav.

[22] Waring ME, Moore Simas TA, Oleski J, Xiao RS, Mulcahy JA, May CN, Pagoto SL (2018). Feasibility and acceptability of delivering a postpartum weight loss intervention via Facebook: a pilot study. J Nutr Educ Behav.

[23] Herring S, Cruice JF, Bennett GG, Davey A, Foster GD (2014). Using technology to promote postpartum weight loss in urban, low-income mothers: a pilot randomized controlled trial. J Nutr Educ Behav.

[24] Silfee VJ, Lopez-Cepero A, Lemon SC, Estabrook B, Nguyen O, Wang ML, Rosal MC (2018). Adapting a behavioral weight loss intervention for delivery via Facebook: a pilot series among low-income postpartum women. JMIR Form Res.

[25] Herring SJ, Bersani VM, Santoro C, McNeil SJ, Kilby LM, Bailer B (2021). Feasibility of using a peer coach to deliver a behavioral intervention for promoting postpartum weight loss in Black and Latina mothers. Transl Behav Med.

[26] Diabetes Prevention Program (DPP) Research Group (2002). The Diabetes Prevention Program (DPP): description of lifestyle intervention. Diabetes Care.

[27] Pagoto S, Waring ME, May CN, Ding EY, Kunz WH, Hayes R, Oleski JL (2016). Adapting behavioral interventions for social media delivery. J Med Internet Res.

[28] Waring ME, Libby BA, Moore Simas TA, Bracken ML, Bibeau JL, Herrera V, Wang J, Pagoto SL (2019). Delivering a post-partum weight loss intervention via Facebook or in-person groups: protocol for a randomized feasibility pilot trial. JMIR Res Protoc.

[29] Harris PA, Scott KW, Lebo L, Hassan N, Lightner C, Pulley J (2012). ResearchMatch: a national registry to recruit volunteers for clinical research. Acad Med.

[30] Cox JL, Holden JM, Sagovsky R (1987). Detection of postnatal depression. Development of the 10-item Edinburgh Postnatal Depression Scale. Br J Psychiatry.

[31] American Psychiatric Association (2013). Diagnostic and Statistical Manual of Mental Disorders (DSM-5). 5th edition.

[32] Moseson H, Fix L, Hastings J, Stoeffler A, Lunn MR, Flentje A, Lubensky ME, Capriotti MR, Ragosta S, Forsberg H, Obedin-Maliver J (2021). Pregnancy intentions and outcomes among transgender, nonbinary, and gender-expansive people assigned female or intersex at birth in the United States: results from a national, quantitative survey. Int J Transgend Health.

[33] Jake-Schoffman DE, Brown SD, Baiocchi M, Bibeau JL, Daubenmier J, Ferrara A, Galarce MN, Hartogensis W, Hecht FM, Hedderson MM, Moran PJ, Pagoto SL, Tsai AL, Waring ME, Kiernan M (2021). Methods-motivational interviewing approach for enhanced retention and attendance. Am J Prev Med.

[34] Revilla M, Ochoa C, Loewe G (2017). Using passive data from a meter to complement survey data in order to study online behavior. Soc Sci Comput Rev.

[35] Dewitt B, Feeny D, Fischhoff B, Cella D, Hays RD, Hess R, Pilkonis PA, Revicki DA, Roberts MS, Tsevat J, Yu L, Hanmer J (2018). Estimation of a preference-based summary score for the patient-reported outcomes measurement information system: the PROMIS®-Preference (PROPr) scoring system. Med Decis Making.

[36] Hanmer J, Dewitt B, Yu L, Tsevat J, Roberts M, Revicki D, Pilkonis PA, Hess R, Hays RD, Fischhoff B, Feeny D, Condon D, Cella D (2018). Cross-sectional validation of the PROMIS-preference scoring system. PLoS One.

[37] Lee CF, Hwang FM, Liou YM, Chien LY (2011). A preliminary study on the pattern of weight change from pregnancy to 6 months postpartum: a latent growth model approach. Int J Obes (Lond).

[38] Onyango AW, Nommsen-Rivers L, Siyam A, Borghi E, de Onis M, Garza C, Lartey A, Baerug A, Bhandari N, Dewey KG, Araújo CL, Mohamed AJ, Van den Broeck J, WHO Multicentre Growth Reference Study Group (2011). Post-partum weight change patterns in the WHO multicentre growth reference study. Matern Child Nutr.

[39] Otten JJ, Hellwig JP, Meyers LD (2006). Dietary Reference Intakes: The Essential Guide to Nutrient Requirements.

[40] Committee on Obstetric Practice (2002). ACOG committee opinion. Exercise during pregnancy and the postpartum period. Number 267, January 2002. American College of Obstetricians and Gynecologists. Int J Gynaecol Obstet.

[41] Chang MW, Nitzke S, Guilford E, Adair CH, Hazard DL (2008). Motivators and barriers to healthful eating and physical activity among low-income overweight and obese mothers. J Am Diet Assoc.

[42] Epifanio MS, Genna V, De Luca C, Roccella M, La Grutta S (2015). Paternal and maternal transition to parenthood: the risk of postpartum depression and parenting stress. Pediatr Rep.

[43] Pagoto SL, Waring ME, Schneider KL, Oleski JL, Olendzki E, Hayes RB, Appelhans BM, Whited MC, Busch AM, Lemon SC (2015). Twitter-delivered behavioral weight-loss interventions: a pilot series. JMIR Res Protoc.

[44] Wang ML, Waring ME, Jake-Schoffman DE, Oleski JL, Michaels Z, Goetz JM, Lemon SC, Ma Y, Pagoto SL (2017). Clinic versus online social network-delivered lifestyle interventions: protocol for the get social noninferiority randomized controlled trial. JMIR Res Protoc.

[45] Food and Nutrition Information Center (U.S.) (2009). Interactive DRI for healthcare professionals. United States Department of Agriculture National Agricultural.

[46] American College of Obstetricians and Gynecologists (2005). ACOG committee opinion number 313, September 2005. The importance of preconception care in the continuum of women's health care. Obstet Gynecol.

[47] Pagoto S, Waring ME, Jake-Schoffman DE, Goetz J, Michaels Z, Oleski J, Di Vito J (2018). What type of engagement predicts success in a Facebook weight loss group?. Proceedings of the 51st Hawaii International Conference on System Sciences.

[48] Smith A (2010). Mobile access 2010. Pew Research Center.

[49] Harris PA, Taylor R, Thielke R, Payne J, Gonzalez N, Conde JG (2009). Research electronic data capture (REDCap)--a metadata-driven methodology and workflow process for providing translational research informatics support. J Biomed Inform.

[50] Frick KD (2009). Microcosting quantity data collection methods. Med Care.

[51] Hernan WH, Brandle M, Zhang P, Williamson DF, Matulik MJ, Ratner RE, Lachin JM, Engelgau MM, Diabetes Prevention Program Research Group (2003). Costs associated with the primary prevention of type 2 diabetes mellitus in the diabetes prevention program. Diabetes Care.

[52] Ritzwoller DP, Sukhanova A, Gaglio B, Glasgow RE (2009). Costing behavioral interventions: a practical guide to enhance translation. Ann Behav Med.

[53] Ramsey SD, Willke RJ, Glick H, Reed SD, Augustovski F, Jonsson B, Briggs A, Sullivan SD (2015). Cost-effectiveness analysis alongside clinical trials II-an ISPOR good research practices task force report. Value Health.

[54] Mertens IL, Van Gaal LF (2000). Overweight, obesity, and blood pressure: the effects of modest weight reduction. Obes Res.

[55] Wing RR, Lang W, Wadden TA, Safford M, Knowler WC, Bertoni AG, Hill JO, Brancati FL, Peters A, Wagenknecht L, Look AHEAD Research Group (2011). Benefits of modest weight loss in improving cardiovascular risk factors in overweight and obese individuals with type 2 diabetes. Diabetes Care.

[56] Leon AC, Davis LL, Kraemer HC (2011). The role and interpretation of pilot studies in clinical research. J Psychiatr Res.

[57] Thabane L, Ma J, Chu R, Cheng J, Ismaila A, Rios LP, Robson R, Thabane M, Giangregorio L, Goldsmith CH (2010). A tutorial on pilot studies: the what, why and how. BMC Med Res Methodol.

[58] Braun V, Clarke V (2006). Using thematic analysis in psychology. Qual Res Psychol.

[59] Groth SW, David T (2008). New mothers' views of weight and exercise. MCN Am J Matern Child Nurs.

[60] Mailey EL, Huberty J, Dinkel D, McAuley E (2014). Physical activity barriers and facilitators among working mothers and fathers. BMC Public Health.

[61] Evenson KR, Aytur SA, Borodulin K (2009). Physical activity beliefs, barriers, and enablers among postpartum women. J Womens Health (Larchmt).

[62] Krukowski RA, Ross KM (2020). Measuring weight with electronic scales in clinical and research settings during the coronavirus disease 2019 pandemic. Obesity (Silver Spring).

[63] Harvey-Berino J, Krukowski RA, Buzzell P, Ogden D, Skelly J, West DS (2011). The accuracy of weight reported in a web-based obesity treatment program. Telemed J E Health.

[64] Jerome GJ, Dalcin A, Coughlin JW, Fitzpatrick S, Wang NY, Durkin N, Yeh HC, Charleston J, Pozefsky T, Daumit GL, Clark JM, Louis TA, Appel LJ (2014). Longitudinal accuracy of web-based self-reported weights: results from the Hopkins POWER Trial. J Med Internet Res.

[65] Caldwell AE, Thomas EA, Rynders C, Holliman BD, Perreira C, Ostendorf DM, Catenacci VA (2022). Improving lifestyle obesity treatment during the COVID-19 pandemic and beyond: new challenges for weight management. Obes Sci Pract.

[66] Cardel MI, Manasse S, Krukowski RA, Ross K, Shakour R, Miller DR, Lemas DJ, Hong YR (2020). COVID-19 impacts mental health outcomes and ability/desire to participate in research among current research participants. Obesity (Silver Spring).

[67] Eysenbach G (2005). The law of attrition. J Med Internet Res.

[68] Pagoto S, Tulu B, Waring ME, Goetz J, Bibeau J, Divito J, Groshon L, Schroeder M (2021). Slip buddy app for weight management: randomized feasibility trial of a dietary lapse tracking app. JMIR Mhealth Uhealth.

[69] Pagoto SL, Schroeder MW, Xu R, Waring ME, Groshon L, Goetz JM, Idiong C, Troy H, DiVito J, Bannor R (2022). A Facebook-delivered weight loss intervention using open enrollment: randomized pilot feasibility trial. JMIR Form Res.

[70] Cavallo DN, Martinez R, Webb Hooper M, Flocke S (2021). Feasibility of a social media-based weight loss intervention designed for low-SES adults. Transl Behav Med.

[71] Han JY, Hou J, Kim E, Gustafson DH (2014). Lurking as an active participation process: a longitudinal investigation of engagement with an online cancer support group. Health Commun.

[72] Cooper P (2021). How Facebook's algorithm works in 2021 and how to make it work for you. One18media.

[73] Pagoto S, Waring ME (2016). A call for a science of engagement: comment on Rus and Cameron. Ann Behav Med.

[74] Franciosi EB, Tan AJ, Kassamali B, Leonard N, Zhou G, Krueger S, Rashighi M, LaChance A (2021). The impact of telehealth implementation on underserved populations and no-show rates by medical specialty during the COVID-19 pandemic. Telemed J E Health.

[75] Fischer SH, Uscher-Pines L, Roth E, Breslau J (2021). The transition to telehealth during the first months of the COVID-19 pandemic: evidence from a national sample of patients. J Gen Intern Med.

[76] Tozour JN, Bandremer S, Patberg E, Zavala J, Akerman M, Chavez M, Mann DM, Testa PA, Vintzileos AM, Heo HJ (2021). Application of telemedicine video visits in a maternal-fetal medicine practice at the epicenter of the COVID-19 pandemic. Am J Obstet Gynecol MFM.

[77] McClain C, Vogels EA, Perrin A, Sechopoulos S, Rainie L (2021). The internet and the pandemic. Pew Research Center.

[78] Ross KM, Carpenter CA, Arroyo KM, Shankar MN, Yi F, Qiu P, Anthony L, Ruiz J, Perri MG (2022). Impact of transition from face-to-face to telehealth on behavioral obesity treatment during the COVID-19 pandemic. Obesity (Silver Spring).

[79] Zaman A, Sloggett KJ, Caldwell AE, Catenacci VA, Cornier MA, Grau L, Vetter C, Rynders CA, Thomas EA (2022). The effects of the COVID-19 pandemic on weight loss in participants in a behavioral weight-loss intervention. Obesity (Silver Spring).

[80] Batsis JA, McClure AC, Weintraub AB, Sette D, Rotenberg S, Stevens CJ, Gilbert-Diamond D, Kotz DF, Bartels SJ, Cook SB, Rothstein RI (2020). Barriers and facilitators in implementing a pilot, pragmatic, telemedicine-delivered healthy lifestyle program for obesity management in a rural, academic obesity clinic. Implement Sci Commun.

[81] Jain B, Bajaj SS, Stanford FC (2022). Randomized clinical trials of weight loss: pragmatic and digital strategies and innovations. Contemp Clin Trials.

[82] Phelan S, Hagobian T, Brannen A, Hatley KE, Schaffner A, Muñoz-Christian K, Tate DF (2017). Effect of an internet-based program on weight loss for low-income postpartum women: a randomized clinical trial. JAMA.

[83] Matthews T, Hamilton BE (2019). Educational attainment of mothers aged 25 and over: United States, 2017. NCHS Data Brief.

[84] Haughton CF, Silfee VJ, Wang ML, Lopez-Cepero AC, Estabrook DP, Frisard C, Rosal MC, Pagoto SL, Lemon SC (2018). Racial/ethnic representation in lifestyle weight loss intervention studies in the United States: a systematic review. Prev Med Rep.

[85] Rosenbaum DL, Piers AD, Schumacher LM, Kase CA, Butryn ML (2017). Racial and ethnic minority enrollment in randomized clinical trials of behavioural weight loss utilizing technology: a systematic review. Obes Rev.

[86] Crane MM, Seburg EM, Levy RL, Jeffery RW, Sherwood NE (2020). Using targeting to recruit men and women of color into a behavioral weight loss trial. Trials.

[87] Cui Z, Truesdale KP, Robinson TN, Pemberton V, French SA, Escarfuller J, Casey TL, Hotop AM, Matheson D, Pratt CA, Lotas LJ, Po'e E, Andrisin S, Ward DS (2019). Recruitment strategies for predominantly low-income, multi-racial/ethnic children and parents to 3-year community-based intervention trials: Childhood Obesity Prevention and Treatment Research (COPTR) consortium. Trials.

[88] Chang MW, Brown R, Nitzke S (2017). Results and lessons learned from a prevention of weight gain program for low-income overweight and obese young mothers: mothers in motion. BMC Public Health.

